# Complete chloroplast genome of *Eucampia zodiacus* (Mediophyceae, Bacillariophyta)

**DOI:** 10.1080/23802359.2021.1944828

**Published:** 2021-07-01

**Authors:** Mengjia Zhang, Zongmei Cui, Feng Liu, Nansheng Chen

**Affiliations:** a CAS Key Laboratory of Marine Ecology and Environmental Sciences, Institute of Oceanology, Chinese Academy of Sciences, Qingdao, China; b Laboratory of Marine Ecology and Environmental Science, Qingdao National Laboratory for Marine Science and Technology, Qingdao, China; c University of Chinese Academy of Sciences, Beijing, China; d Center for Ocean Mega-Science, Chinese Academy of Sciences, Qingdao, China; e Department of Molecular Biology and Biochemistry, Simon Fraser University, Burnaby, Canada

**Keywords:** Diatoms, chloroplast genome, *Eucampia zodiacus*

## Abstract

The cosmopolitan phytoplankton species *Eucampia zodiacus* Ehrenberg 1839 is a common harmful algal bloom (HAB) species with significant negative ecological impact. However, molecular information for this HAB species is limited. In this study, the complete chloroplast genome (cpDNA) of *E. zodiacus* was constructed for the first time. The circular genome was 118,107 bp in length, containing a pair of inverted repeats (IR) (6991 bp each). The overall GC content of *E. zodiacus* cpDNA was 31.64%. It encoded 169 genes, including 131 protein-coding genes (PCGs), 30 tRNA genes, one tmRNA gene, one ncRNA gene, and six rRNA genes in IR regions. Phylogenetic analysis using concatenated PCGs of 56 diatom cpDNAs strongly supported that *E. zodiacus* was closely related to *Cerataulina daemon*, which belongs to the same family Hemiaulaceae according to AlgaeBase. Syntenic analysis revealed nearly identical gene order between the two cpDNAs, except for an inversion in the small single-copy (SSC) region.

*Eucampia zodiacus* Ehrenberg 1839 is a common harmful algal bloom (HAB) species with a worldwide distribution except for the polar regions. It can be detected almost all-year round in the water column, providing considerable primary production (Horner 2002; Ito et al. 2013; Nishikawa et al. 2013). *Eucampia zodiacus* can form dense blooms in coastal waters, which have been observed in many countries including Japan, Canada and China (Huo et al. 2001; Zhang et al. 2002; Nishikawa et al. 2007; Martin et al. 2008; Nishikawa et al., 2011; Liang 2012; Matsubara 2012). *E. zodiacus* blooms have been reported to cause bleaching of aquacultured nori, damages to fisheries and economic losses (Martin et al. 2008; Nishikawa et al., 2011). Despite of its important role in environment and ecology, molecular information for this HAB species is limited. In recent study, we have constructed the first mitochondrial genome (mtDNA) of *E. zodiacus*, and through the comparative analysis, we defined a high-resolution molecular marker for tracking its genetic diversity (Zhang et al. 2021). In this project, we constructed the chloroplast genome (cpDNA) of *E. zodiacus* for the first time, and submitted the annotated genomic sequence to GenBank under accession number MW412838.

The *E. zodiacus* strains CNS00061 was isolated from seawater samples collected from the Changjiang Estuary (30.36°N, 122.86°E) (July, 2019). The specimen was deposited in the collection of marine algae in KLMEES of IOCAS (Nansheng Chen, chenn@qdio.ac.cn) under the voucher number CNS00061. The strain was confirmed as *E. zodiacus* both through morphological (Guo 2004; Yang and Dong 2006) and molecular identification using five common molecular markers, including full-length 18S rDNA (Sorhannus 2007), 28S rDNA D1–D2 region (Hamsher et al. 2013), ITS (Guo et al. 2015), *COI* (Guo et al. 2015) and *rbcL* (Guo et al. 2015). Genomic DNA was extracted with TIANGEN DNAsecure Plant Kit (TIANGEN, DP121221) and sequenced on the Illumina Novaseq 6000 platform at Novogene (Beijing, China), generating 150 bp paired-end reads from libraries 350 bp in length.

The filtered reads were assembled into scaffolds with Platanus-allee (v2.2.2) (Kajitani et al. 2019) and SPAdes (v3.14.0) (Bankevich et al. 2012). With the cpDNA of *Cerataulina daemon* (KJ958484) (Sabir et al. 2014) used as the reference, scaffolds corresponding to cpDNA of *E. zodiacus* were screened using BLASTN. To fill the gap, Sanger sequencing was used to extend the ends of scaffolds and the gap was covered twice to ensure the accuracy. Assembly errors were corrected and N regions were replaced by using BWA (v0.7.17–r1188) (Li and Durbin 2009), results of which were extracted with SAMtools (v1.10) (Li et al. 2009) and viewed with IGV (v2.7.2) (Robinson et al. 2011). Protein-coding genes (PCGs) and open reading frames (*orfs*) were annotated using NCBI ORF Finder and BLAST similarity searches of the non-redundant databases at NCBI (Altschul et al. 1997). tRNAs were determined by reconstructing their cloverleaf structures using the tRNAscan-SE (v1.3.1) (Lowe and Chan 2016). rRNAs were identified using RNAmmer (v1.2) (Lagesen et al. 2007), Barrnap (v0.9) and MEGA (v7.0) for homologous comparison. The gene map of the circular cpDNA of *E. zodiacus* was generated with Organellar Genome DRAW (OGDraw) (Lohse et al. 2007).

The complete cpDNA of *E. zodiacus* (strain CNS00061) was a circular molecule that was 118,107 bp in size. The whole cpDNA contained a pair of inverted repeats (IR) regions of 6,991 bp in length. The IR regions divided the genome into two single-copy regions, small single copy (SSC) and large single copy (LSC) with 38,079 and 66,046 bp, respectively. The GC contents of the LSC, SSC, IR regions and the cpDNA as a whole, are 30.37%, 30.57%, 40.58%, and 31.64%, respectively. It possessed 169 genes, out of which, there were 131 PCGs, 30 tRNA genes, one tmRNA, one ncRNA, and six rRNA genes (three rRNA species). Among the 131 PCGs, 129 genes started with the canonical ATG start codons, *rpl23* with GTG and *rbcS* with ATA. No introns were found in the *E. zodiacus* cpDNA.

To confirm the phylogenetic position of *E. zodiacus* within the phylum Bacillariophyta, the amino acid (aa) sequence dataset of 95 concatenated PCGs of 56 diatom species was obtained to construct phylogenetic tree. *Triparma laevis* (AP014625) (Bolidophyceae, Ochrophyta) was used as out-group taxa. Maximum likelihood (ML) phylogenetic tree (Figure 1) constructed using IQ-TREE (v1.6.12) (Trifinopoulos et al. 2016) with 1000 bootstrap replicates demonstrated that the 56 species in Bacillariophyta formed six clades in three classes, Coscinodiscophyceae, Mediophyceae, and Bacillariophyceae. Bacillariophyceae (‘Clade 1’) was recovered as monophyletic, within which *Astrosyne radiata* was recovered on an extremely long branch. Mediophyceae, which consisted of three clades (‘Clade 2’, ‘Clade 3’ and ‘Clade 6’), was not monophyletic. Among these three clades, *Attheya longicornis* and two *Biddulphia* species formed Clade 2, and were sister to the Bacillariophyceae diatoms (‘Clade 1’) with 100% bootstrap support. Furthermore, the species *Leptocylindrus danicus*, which as annotated at AlgaeBase as a species in the class Mediophyceae, did not cluster together with other species of this class. Coscinodiscophyceae was not monophyletic, including two clades (‘Clade 4’ and ‘Clade 5’). The species *Proboscia* sp. (‘Clade 4’), which was annotated as a species in the class Coscinodiscophyceae, did not cluster together with the species of this class. This result was consistent to findings reported previously (Yu et al. 2018).

**Figure 1.**
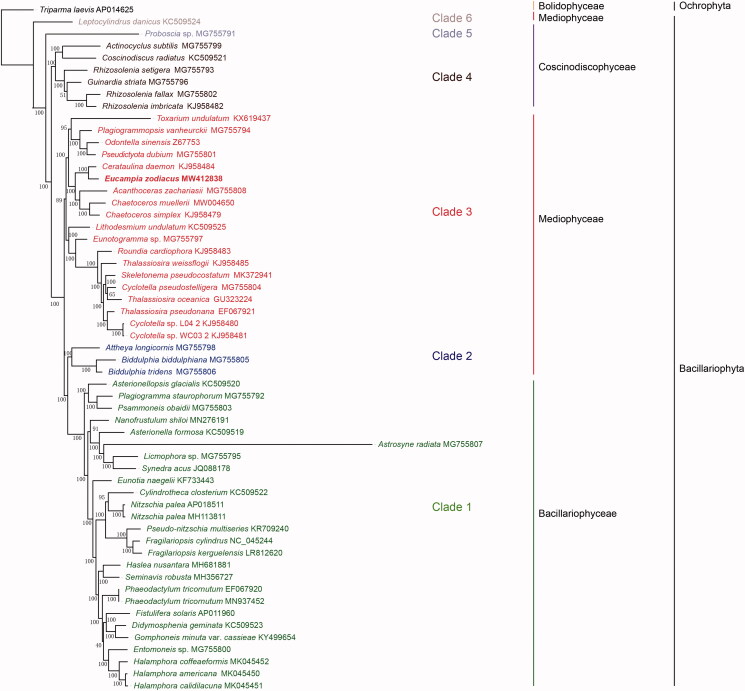
Phylogenetic tree based on ML analysis of aa sequence dataset of 95 cpDNA PCGs in Bacillariophyta. *Triparma laevis* (AP014625) (Bolidophyceae, Ochrophyta) was used as out-group taxa. Numbers on the branches represent bootstrap values.

*E. zodiacus* was grouped together with *C. daemon*, which was consistent to the current classification in AlgaeBase. Furthermore, syntenic analysis between the cpDNAs of *E. zodiacus* and *C. daemon* revealed nearly identical gene order, except for an inversion in the SSC region, which is a well-accepted feature of cpDNAs (Doyle, 1999). The orientation of the SSC region may be different between these two species, due to evolutionary changes. However, we could not rule out the possibility that the two species have heteroplasmy (Walker et al. 2015), which remains to be confirmed by further analyses. Compared to *C. daemon*, cpDNA of *E. zodiacus* also lacked *syfB* gene, which is absent from all species in another order Thalassiosirales (Mediophyceae) (Sabir et al. 2014). The cpDNAs of more species in the order Hemiaulales are needed to track gene loss and clarify the evolutionary relationships between two orders.

## Data Availability

The genome sequence data that support the findings of this study are openly available in GenBank of NCBI at https://www.ncbi.nlm.nih.gov/nuccore/MW412838, under the accession no. MW412838. The associated BioProject, SRA and Bio-Sample numbers are PRJNA682714 (https://www.ncbi.nlm.nih.gov/bioproject/PRJNA682714), SRR13201560 (https://www.ncbi.nlm.nih.gov/sra/SRR13201560) and SAMN17005956 (https://www.ncbi.nlm.nih.gov/biosample/SAMN17005956), respectively.
